# Supplementary material for: Functional organization and natural scene responses across mouse visual cortical areas revealed with encoding manifolds
Source: PLoS One. 2026 Sep 17;21(9):e0356243. doi: 10.1371/journal.pone.0356243 (PMC13585280; doi:10.1371/journal.pone.0356243)
Supplement: Supplementary file 1 [file pone.0356243.s019.pdf]

S1 Table. Effect sizes and statistical significance for layer-specific natural-scene vs grating preference.

Area	Layer	Method	n	Cohen's d	p_{FDR}	Sig.
VISal	L2/3	max	71	0.423	3.51e-03	Yes
VISal	L2/3	mean	71	0.229	1.12e-01	No
VISal	L4	max	116	0.177	1.12e-01	No
VISal	L4	mean	116	-0.139	2.33e-01	No
VISal	L5	max	191	0.103	2.59e-01	No
VISal	L5	mean	191	-0.113	2.12e-01	No
VISal	L6	max	49	-0.194	2.82e-01	No
VISal	L6	mean	49	-0.325	6.93e-02	No
VISam	L2/3	max	120	0.304	5.55e-03	Yes
VISam	L2/3	mean	120	-0.0139	9.38e-01	No
VISam	L4	max	83	0.265	5.08e-02	No
VISam	L4	mean	83	-0.0673	6.66e-01	No
VISam	L5	max	211	-0.0446	6.54e-01	No
VISam	L5	mean	211	-0.198	1.90e-02	Yes
VISam	L6	max	64	-0.206	1.92e-01	No
VISam	L6	mean	64	-0.329	3.65e-02	Yes
VISl	L2/3	max	35	0.485	2.59e-02	Yes
VISl	L2/3	mean	35	0.0206	9.43e-01	No
VISl	L4	max	49	0.626	6.15e-04	Yes
VISl	L4	mean	49	0.0849	6.66e-01	No
VISl	L5	max	151	0.192	5.29e-02	No
VISl	L5	mean	151	-0.0684	5.36e-01	No
VISl	L6	max	45	-0.0313	9.11e-01	No
VISl	L6	mean	45	-0.368	5.08e-02	No
VISp	L2/3	max	100	0.633	1.74e-07	Yes
VISp	L2/3	mean	100	0.52	1.30e-05	Yes
VISp	L4	max	141	0.663	4.01e-11	Yes
VISp	L4	mean	141	0.441	9.25e-06	Yes
VISp	L5	max	227	0.186	2.25e-02	Yes
VISp	L5	mean	227	-0.0471	6.21e-01	No
VISp	L6	max	117	-0.356	1.53e-03	Yes
VISp	L6	mean	117	-0.342	2.28e-03	Yes
VISpm	L2/3	max	45	0.206	2.80e-01	No
VISpm	L2/3	mean	45	0.00542	9.71e-01	No
VISpm	L4	max	28	0.411	8.44e-02	No
VISpm	L4	mean	28	0.0868	7.43e-01	No
VISpm	L5	max	97	0.102	4.55e-01	No
VISpm	L5	mean	97	-0.026	8.91e-01	No
VISpm	L6	max	25	-0.513	5.08e-02	No
VISpm	L6	mean	25	-0.45	8.13e-02	No
VISrl	L2/3	max	69	0.144	3.52e-01	No
VISrl	L2/3	mean	69	0.0672	6.77e-01	No
VISrl	L4	max	131	0.313	2.85e-03	Yes
VISrl	L4	mean	131	-0.00762	9.50e-01	No
VISrl	L5	max	115	-0.195	8.44e-02	No
VISrl	L5	mean	115	-0.188	9.74e-02	No
VISrl	L6	max	34	-0.168	4.57e-01	No
VISrl	L6	mean	34	-0.172	4.55e-01	No
